# The Role of the Adipokines in the Most Common Gestational Complications

**DOI:** 10.3390/ijms21249408

**Published:** 2020-12-10

**Authors:** Paweł Gutaj, Rafał Sibiak, Maurycy Jankowski, Karina Awdi, Rut Bryl, Paul Mozdziak, Bartosz Kempisty, Ewa Wender-Ozegowska

**Affiliations:** 1 Department of Reproduction, Chair of Obstetrics, Gynecology, and Gynecologic Oncology, Poznań University of Medical Sciences, 60-535 Poznan, Poland; 75094@student.ump.edu.pl (R.S.); ewaoz@post.pl (E.W.-O.); 2 Department of Histology and Embryology, Poznań University of Medical Sciences, 60-781 Poznan, Poland; bkempisty@ump.edu.pl; 3 Department of Anatomy, Poznań University of Medical Sciences, 60-781 Poznan, Poland; mjankowski@ump.edu.pl (M.J.); rutbryl@gmail.com (R.B.); 4 Student’s Scientific Society, Poznan University of Medical Sciences, 60-806 Poznan, Poland; avdikarina@gmail.com; 5 Physiology Graduate Program, North Carolina State University, Raleigh, NC 27695-7608, USA; pemozdzi@ncsu.edu; 6 Department of Obstetrics and Gynecology, University Hospital, Masaryk University, 625 00 Brno, Czech Republic; 7 Department of Veterinary Surgery, Institute of Veterinary Medicine, Nicolaus Copernicus University in Toruń, 87-100 Torun, Poland

**Keywords:** adiponectin, apelin, chemerin, gestational diabetes, irisin, leptin, omentin, preeclampsia, resistin, visfatin

## Abstract

Adipocytokines are hormonally active molecules that are believed to play a key role in the regulation of crucial biological processes in the human body. Numerous experimental studies established significant alterations in the adipokine secretion patterns throughout pregnancy. The exact etiology of various gestational complications, such as gestational diabetes, preeclampsia, and fetal growth abnormalities, needs to be fully elucidated. The discovery of adipokines raised questions about their potential contribution to the molecular pathophysiology of those diseases. Multiple studies analyzed their local mRNA expression and circulating protein levels. However, most studies report conflicting results. Several adipokines such as leptin, resistin, irisin, apelin, chemerin, and omentin were proposed as potential novel early markers of heterogeneous gestational complications. The inclusion of the adipokines in the standard predictive multifactorial models could improve their prognostic values. Nonetheless, their independent diagnostic value is mostly insufficient to be implemented into standard clinical practice. Routine assessments of adipokine levels during pregnancy are not recommended in the management of both normal and complicated pregnancies. Based on the animal models (e.g., apelin and its receptors in the rodent preeclampsia models), future implementation of adipokines and their receptors as new therapeutic targets appears promising but requires further validation in humans.

## 1. Introduction

According to the newest data, 30.5% of European women are overweight (body mass index (BMI) > 25 kg/m^2^), and a stunning 15.9% of them are diagnosed with obesity (BMI > 30 kg/m^2^), which forces obstetricians to face with the state of an obesity epidemic and obesity-related diseases among their patients [1]. Moreover, the pregnancy itself is connected with increased insulin resistance and promotes the worsening of multiple metabolic parameters. The exact pathophysiological mechanism of numerous pregnancy complications such as gestational hypertension, preeclampsia (PE), gestational diabetes (GDM), or fetal growth abnormalities is quite elaborate, however, its incidence among obese patients is significantly elevated [2,3,4].

Obesity is a pathological condition considered to be mainly associated with bad lifestyle habits—high caloric intake and the lack of physical activity—leading to morphological changes in the structure of adipose tissue [5]. Excessive body weight promotes adipocyte hypertrophy and the occurrence of the local cellular hypoxia, which together contribute to the development of the state of chronic low-grade inflammation [6]. This results in increased migration and the presence of immune cells, such as macrophages and lymphocytes, in pathologically re-modelled adipose tissue due to increased adipocyte apoptosis promoted by hypoxia [7]. The increased infiltration of immune cells exposed to persistent hypoxia and increased oxidative stress destroys the balance between secretion of anti- and pro-inflammatory cytokines [8]. In response to the presence of those stressors, the synthesis of various adipocyte-specific molecules, such as leptin and adiponectin, is altered compared to lean individuals. It has been found that adipokine secretion patterns are strictly linked with the excessive body weight, percentage of hormonally active adipose tissue, and inflammatory status [9]. However, these changes can be reversed through a reduction of both subcutaneous and visceral fat levels [10,11]. 

Adipokines belong to the group of protein hormones and cytokines secreted by adipocytes, immune cells, fibroblasts, and other hormone-secreting cells originating from the adipose tissue. Adipocytes release hundreds of signaling molecules that are responsible for the regulation of the local and systemic cellular activities through their autocrine, paracrine, and endocrine activity [12,13]. It has been established that adipokines play a key role in the regulation of many crucial processes in the human body, like glucose and lipid metabolism, insulin sensitivity, appetite, immune response, and inflammation [14]. Due to their engagement in these processes, adipokines may be treated as potential targets for novel therapeutic strategies in numerous medical conditions [15].

A dysregulation in adipokine production is known as a characteristic feature of obesity and occurs in a variety of obesity-related diseases. Furthermore, those abnormalities are treated as partial causative factors in the pathogenesis of several metabolic, inflammatory, and neoplastic diseases [16]. The abnormal leptin-to-adiponectin ratio is often observed in test results of individuals diagnosed with impaired glucose tolerance, dyslipidemias, and high blood pressure [17,18]. Without any predominant changes in daily habits and diet, those conditions lead straight to the development of the metabolic syndrome [19]. Patients affected by metabolic syndrome are often diagnosed with type 2 diabetes and advanced atherosclerosis, which makes them more susceptible to micro- and macro-vascular diseases and puts them at risk of death from cardiovascular events like stroke or heart attack [20].

While there are still multiple possible hypotheses regarding the mechanisms of pregnancy-related complications, such as gestational hypertension, preeclampsia, gestational diabetes, or fetal growth abnormalities, the discovery of the adipose-derived hormones sheds new light on those conditions. Current studies tried to analyze the contribution of adipocyte-specific peptides to the pathogenesis of those complications. Moreover, it seems essential to assess the possible benefits of routine adipokines concentration measurements performed in patients with uncomplicated pregnancies. Those assessments could establish their utility as potential early markers of gestational complications. Standard measurements of adipokine levels in a few mentioned high-risk pregnancy complications may also influence the choice of proper treatment strategies. Finally, adipokines open promising perspectives for the invention of new drugs that could be used during gestation.

## 2. Scope and Methodology

This review was compiled to analyze the potential clinical applications of adipokine measurements in early detection and further management strategies of a variety of gestational complications. A list of analyzed adipokines was restricted to relatively well-characterized molecules that included leptin, adiponectin, visfatin, resistin, irisin, omentin, chemerin, and apelin. Every adipokine is characterized in a separate section. Gestational complications were limited to those which are thought to be even partially associated with disturbances in patient’s metabolic status—maternal complications and fetal abnormalities that might be related to maternal metabolic diseases. Analyzed conditions include obesity, gestational diabetes, gestational hypertension, preeclampsia, fetal growth restriction (FGR), and fetal macrosomia. To establish a list of relevant references, PubMed database was searched from the first records until September 2020 using the following formulas: (“an adipokine” + “a pregnancy complication”)—from those listed above.

## 3. Clinical Applications of Adipokines Measurements in Various Pathologies

### 3.1. Leptin

Leptin is a peptide hormone secreted mainly by the white adipose tissue, with its most important functions including regulation of energy homeostasis, metabolism, and neuroendocrine function [21]. Leptin was reported to be elevated even in physiological pregnancy, steadily increasing throughout its course [22]. Leptin was the first described adipokine linking the adipose tissue with reproduction [23]. This protein is also expressed by the placental tissue and fetal adipose tissue. Ninety-five percent of leptin produced in the placenta is delivered into maternal circulation where it promotes excessive weight gain during pregnancy [24,25,26]. Hence, leptin is often considered to be responsible for pregnancy-associated energy balance changes [27]. Furthermore, reproductive functions, such as embryo implantation and development, have also been found to be influenced by its expression. In turn, mouse model studies reported infertility caused by leptin deficiency, with the affected animals rescued by exogenous administration of this protein [28]. Leptin was also found to stimulate gonadotropin hormone expression, as well as act as a puberty permissive factor under the influence of steroid hormones [29]. It was further reported to affect ovarian function, linking follicular alterations with obesity [30]. Additionally, a significant role of leptin was suggested in embryo implantation, as the protein and its receptors are expressed in blastocysts and can be extracted from embryo-conditioned media [31,32].

#### 3.1.1. Gestational Diabetes Mellitus (GDM)

**Figure 1:**
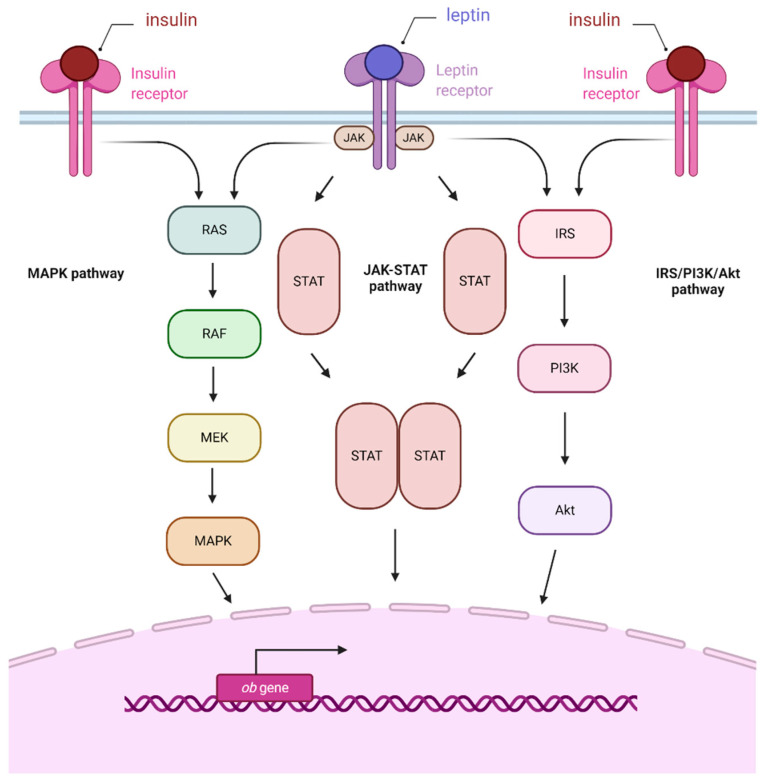
Pictorial diagram of insulin and leptin signaling in placenta which includes 3 major signaling pathways, namely JAK-STAT, MAPK, and IRS/PI3K/Akt. Abbreviations: Akt—protein kinase B; IRS—insulin receptor substrate; JAK—janus kinase; MAPK—mitogen-activated protein kinase; MEK—mitogen-activated protein kinase kinase; PI3K—phosphatidylinositol 3-kinase; RAF—RAF kinase; RAS—small GTPase; STAT—signal transducer and activator of transcription (according to References [38,39]) (Created with BioRender.com).

GDM is defined as maternal hyperglycemia and glucose intolerance of varying degrees of severity which occur for the first time during pregnancy. The diagnosis is based on fasting glucose and oral glucose tolerance test results [4]. GDM patients’ placentas were characterized by increased expression of leptin and leptin receptors, with this protein suggested as a first-trimester prognostic factor for this condition [33]. Furthermore, leptin was suggested to control fetal homeostasis in response to hyperinsulinemia [34,35]. Cord blood leptin levels also seem to be dependent on maternal glucose, providing a possible explanation to the predisposition of GDM newborns to obesity [36]. Moreover, increased leptin gene expression in GDM placentas compared to controls correlated with pro-inflammatory cytokine production, resulting in chronic inflammation and further amplification of leptin production [37]. It was reported that insulin induces leptin expression in cells of the trophoblast through an increase in activity of leptin promoter, as those two hormones share several signaling pathways (e.g., MAPK, PI3K, and JAK2/STAT-3) [38]. Moreover, a placental crosstalk between leptin and insulin signaling was demonstrated, manifesting through increased STAT-3, MAPK 1/3, and PKB pathway activity (Figure 1) [39]. A study evaluating plasma levels of leptin also showed its increase in plasma of patients with GDM [40]. This was confirmed in further studies, which showed a statistically significant increase of venous blood and serum levels of this protein in women affected by GDM compared to healthy controls [22,40]. These results were confirmed in a recent publication, which furthermore suggests that while the overall baseline concentration of circulating leptin is higher in GDM patients, a smaller increase in the levels of this protein can be observed throughout the course of pregnancy [22].

Overall, while the available studies suggest that there is an increase in leptin levels in GDM patients compared to controls, possible explanations and implications of that occurrence are not sufficiently explored. Hence, there is an understandable lack of clinical studies targeting leptin in the treatment of GDM. 

#### 3.1.2. Preeclampsia

The American College of Obstetrics and Gynecology defines PE as a pregnancy complication which occurs after 20 weeks of gestation. The current criteria of PE includes elevated blood pressure with the presence of other severe features with or without proteinuria [41], and in severe cases, widespread organ failure. It is a major contributor to maternal as well as fetal morbidity and mortality [41]. The etiology of PE is probably heterogeneous and not completely clarified, but it is believed that the disease starts in the first trimester as a placental condition resulting in insufficient placental perfusion, followed by a maternal syndrome with endothelial dysfunction and hypertension [42].

Multiple studies suggest an increase in leptin serum levels in PE patients, as well as elevated levels of this protein in preeclamptic placentas [43,44,45]. However, the same correlation was not observed in pre-term PE [45]. Hence, while the connection between BMI and PE has not yet been defined, leptin has been among the proposed factors that could facilitate that link [45]. Two suggested dependencies either indicated leptin increase as a sign of placental stress, such as preeclampsia-associated hypoxia, or note its increase even before the onset of the disease, with the possibility of its use as a prognostic factor [46]. Another explanation, basing on the proangiogenic ability of leptin, suggests its role in response to under-perfusion of PE placenta, supporting neovascularization and improving nutrient delivery [37].

Overall, while the mode of leptin action during preeclampsia is not yet fully determined, multiple studies agree that its levels are elevated in PE patients [21]. Hence, this protein could potentially become a diagnostic-prognostic factor in this condition. However, a recent study reported about decreased levels of this protein in PE-affected obese women compared to normal pregnancy controls [47].

#### 3.1.3. Other Gestational Complications

Apart from PE and GDM, the role of leptin in recurrent miscarriages is an important topic [48]. Women affected by recurrent miscarriage exhibited elevated serum leptin concentration compared to the control group [49]. However, in another study, 5–8 gestation weeks plasma levels in women who subsequently miscarried were found to be lower than among women in which term birth was noted [50]. Finally, the study of Tommaselli et al. found no significant difference in maternal serum leptin concentration, maybe due to the heterogeneity of the miscarriage cases in the study group [51]. Hence, the data on leptin concentration in recurrent miscarriage are highly conflicted, with no consensus that could shed light on the possible involvement of this protein in this condition.

Cord blood leptin levels are positively correlated with neonatal birth weight. Moreover, large-for-gestational-age (LGA) newborns have higher cord blood leptin concentrations in comparison to appropriate-for-gestational-age controls. Thus, it was established that elevated leptin levels promote the increased fetal weight gain [52,53]. Intrauterine growth restriction (IUGR) was also associated with decreased leptin levels in both maternal and fetal blood. While the detected levels of both were lower in IUGR pregnancies, there was no statistically significant relationship between the maternal and fetal concentrations [54]. Furthermore, a comparison between PE and IUGR patients (without PE) showed that the levels of leptin were significantly lower in patients without preeclampsia, showing differences despite similar placental pathology [55]. 

### 3.2. Adiponectin

Adiponectin is a 30 kDa adipokine most abundantly secreted by adipocytes, with reported antidiabetic, anti-inflammatory, and cardioprotective functions [56]. Its expression was shown to exhibit significant sexual dimorphism, with males characterized by notably lower levels of this protein than females [57], while its action is exerted through two functionally different receptors, AdipoR1 and AdipoR2. It is one of the hormones that were, in a number of studies, associated with the development of obesity [58]. Its reduction plays a significant role in diseases associated with excessive weight, as it counteracts insulin resistance and inflammation [59]. Hence, administration of recombinant adiponectin was a subject of a number of therapeutic interventions [60]. Furthermore, adiponectin is one of the most studied adipokines within the context of pregnancy. It was found that adiponectin, independently from maternal BMI, modulates the glucose homeostasis, promoting the increased insulin sensitivity [61,62]. Most studies suggest that in normal pregnancies, plasma levels of this protein did not significantly differ between non-pregnant and first trimester pregnant women, decreasing towards the end of pregnancy and falling again postpartum [63]. This inverse correlation with the gestational age of the fetus is not observed in overweight women. This dependency can be explained with the need for maternal pregnancy-related fat deposition and the downregulation of adiponectin expression in response to increasing adipose mass [64,65,66,67]. Hence, despite that singular studies based on a small size sample group question the correlation of adiponectin level with gestation advancement, the general consensus states that concentration of this protein decreases significantly between the first and third trimester of pregnancy. Nevertheless, the physiological range of this protein’s serum levels during gestation is not yet established [63]. Finally, adiponectin was implicated in a range of other diseases, such as hypertension, chronic kidney diseases, atherosclerosis, chronic obstructive pulmonary disease, diabetic retinopathy and cancer, as summarized by a number of existing excellent reviews focused solely on this adipokine [59,60].

#### 3.2.1. Gestational Diabetes Mellitus

Adiponectin levels are decreased in GDM patients, regardless of their weight, BMI, or insulin resistance [37,68,69,70,71]. Moreover, some studies suggest that adiponectin pre-pregnancy and early pregnancy measurements could act as a predictive factor for the later development of GDM, with some of them including a combination of other maternal factors to improve prediction rate [72,73,74,75,76]. The results of a meta-analysis conducted by Ilidromiti et al. suggest that in combination with maternal characteristics, blood adiponectin, as a known predictor of insulin resistance status, could indeed be used to evaluate the risk of GDM [77]. Furthermore, decreased adiponectin mRNA levels were also detected in placental tissue of GDM patients [78]. A decrease in adiponectin levels can also be observed in circulation of fetuses born out of GDM pregnancies, compared to the controls of the same gestational age, independent of the fetal birth weight [79]. 

Overall, the results of adiponectin studies are relatively uniform, with the levels of this protein in maternal blood generally accepted as a good potential prognostic/diagnostic factor for GDM.

#### 3.2.2. Preeclampsia

Adiponectin receptor’s mRNA and protein are upregulated in preeclampsia, while adipokine itself shows downregulation in placental tissue [80,81]. These levels were also found to correlate positively with the expression of p-STAT5 and inversely with p-p38 (both factors affecting the function of placental trophoblasts), implicating the role of adiponectin in preeclampsia [82]. Ramsay et al. found a significant rise in adiponectin concentration in patients affected by PE compared to controls, suggesting that this increase is tied to higher adipocyte secretion [83]. These results were confirmed by multiple later studies, even those correcting for hematocrit [84,85,86,87]. However, several different studies reported significantly lower levels of circulating adiponectin in preeclamptic patients compared to normal pregnancy [88,89,90]. A proposed explanation of this status is the BMI differences of patients, as those with BMI > 25 and preeclampsia exhibited significantly decreased blood concentration of adiponectin. Similarly, normal-weight woman affected with the same disease showed a major increase in circulating levels of this protein [91]. However, it must be noted that this correlation might be non-functional in overweight women affected by preeclampsia, due to their increased insulin and adiponectin resistance, while in the same way representing the physiological response of the normal-weight women to impaired placental perfusion [64]. This assumption is supported by a recent study published by Thagaard et al., that compared normal and preeclamptic obese pregnant women, confirming that the former were characterized by a significantly lower expression of adiponectin, suggesting that lack of this protein might play a role in the pathogenesis of the disease [47]. Moreover, a recent study evaluating the usefulness of serum adiponectin quantification for prediction of PE concluded that adiponectin levels were the best prognostic factor (as compared to visfatin, resistin, and leptin), when corrected for BMI, age, parity, and family history of PE and diabetes [92]. In summary, the mechanism of adiponectin action in preeclampsia is not yet well known, but it appears that lower levels of this protein might play a certain role in overweight and obese women, with a less likely involvement in normal weight pregnancy.

### 3.3. Visfatin

Visfatin is a protein produced predominantly by the visceral adipose tissue, with its tissue and circulation levels significantly increasing in obesity [93]. Visfatin levels are, also, higher in pregnant compared to non-pregnant controls [94]. During normal pregnancy, the plasma concentration of this protein changes, reaching a peak between 19 and 26 weeks of gestation. In normal pregnancies of obese women, visfatin levels were unchanged [95]. Decreased placental visfatin expression was associated with poor glycemic control and macrosomia in women with type 1 diabetes [96]. However, the physiological range of visfatin in normal pregnancy remains undetermined, with the variation between studies mostly dependent on the characteristics of the studied group [97].

#### 3.3.1. Gestational Diabetes Mellitus

Similarly, in the case of GDM, the results concerning visfatin levels in affected and control patients are conflicting [98]. Akturk et al. in their study of plasma visfatin levels of women between 33 and 39 weeks of gestation, concluded that the concentration of this protein drops in GDM patients compared to healthy controls [99]. Several other studies, on the contrary, reported significantly elevated visfatin levels in GDM pregnancies [100,101,102]. A meta-analysis of 26 original studies conducted by Zhang et al. concluded that the level of this protein is significantly increased in obese mothers affected by GDM, in comparison to controls. However, they also noted that this finding is most probably associated with extreme maternal weight, rather than with GDM itself [103]. Moreover, Mazaki-Tovi et al. determined that maternal GDM and fetal hypertrophy are independently associated with high plasma visfatin levels, indicating its potential usefulness as a diagnostic/prognostic factor for GDM screening [104]. Another study positively correlated umbilical cord visfatin levels with fetal birth weight, indicating its potential role in fetal growth regulation [105]. Taraqi et al. found an inverse correlation between visfatin levels and birth weight in patients with higher than normal BMI [106]. Furthermore, higher levels of this protein were associated with IUGR (defined as birth weight ≤ 3rd customized centile), especially during the third trimester of pregnancy [107,108].

#### 3.3.2. Preeclampsia

The visfatin levels in preeclampsia remain relatively unknown [70]. Fasshauer et al. reported increased serum levels of visfatin in PE patients compared to healthy controls [109]. On the contrary, Hu et al. reported a notable decrease in the levels of this protein among pregnant women with and without PE in the third trimester of pregnancy [110]. The differing results among several studies may be related to the characteristics of the study groups [111,112,113]. Nevertheless, a recent study, evaluating the usefulness of a number of adipokines in PE prediction, concluded that while visfatin levels (reported as higher in PE patients than in controls) are a weaker predictor than, e.g., adiponectin, it was, nonetheless, a significant prognostic factor when corrected for BMI [92].

A significant number of studies tie visfatin to normal and pathological pregnancies, but their results are often conflicting. Furthermore, the gaps in knowledge about the exact function of this protein in physiological, as well as pathological pregnancy, calls for more research before it can be fully recommended as a diagnostic/prognostic factor [98].

### 3.4. Resistin

Resistin is a pro-inflammatory adipokine that is predominantly expressed by mononuclear cells, as well as adipocytes and most importantly, placental trophoblastic cells during pregnancy [114,115]. Resistin impairs glucose uptake by adipocytes, increases plasma glucose concentration, and thus decreases insulin sensitivity [114]. It is also known as a factor inducing the production of inflammatory cytokines and cell adhesion molecules [116]. The plasma resistin levels in pregnant women at term are significantly higher than those in age-matched non-pregnant controls. Because, adipose resistin expression remains unchanged during pregnancy, it suggests that the placenta is likely a major source of resistin in the maternal circulation [79,115]. Moreover, high resistin level could further affect the placental transfer of glucose by decreasing the expression of trophoblastic cell surface glucose transporters, GLUT-1 [117]. Some authors linked the high resistin concentrations with the increase in insulin resistance during the latter half of pregnancy, which may suggest its potential indirect role in the regulation of fetal growth. Those effects could be caused by increased insulin secretion and its pro-growth activity [118].

#### 3.4.1. Gestational Diabetes Mellitus

Even though experimental findings suggest that resistin might be involved in the diabetes pathophysiology and glucose homeostasis, its role in the development of insulin resistance and GDM in pregnancy remains unclear [119,120]. Despite the fact that several studies attempted to assess the concentrations of circulating resistin in GDM, there appears to be a significant discrepancy between the results. It is concluded by most studies that the differences in concentrations between women with GDM and pregnant controls are insignificant [75]. Besides, three independent prospective studies did not find any association between resistin and increased GDM risks [75,76,121]. However, significant differences in levels depending on trimesters have been reported by other studies. Chen et al. and Nien et al. reported that resistin level remains stable in the first and second trimesters and increases in the third trimester [122,123]. In contrast, Cortelazzi et al. found a progressive decline in resistin level between 10 and 41 weeks [79]. However, there was the substantial heterogeneity between the studies. Lobo et al. in their study revealed that significantly higher plasma resistin levels, between 11 and 13 weeks of pregnancy, existed in participants, who later developed GDM as compared to their healthy control counterparts [120].

Interestingly, even though Lappas et al. did not detect any characteristic differences in the release of resistin while comparing GDM patients with pregnant controls, the study showed that several inflammatory mediators and hormones modulate resistin release, including insulin which stimulates the release of resistin from the human placenta [124]. These controversial results may be attributed to not correlating different gestational ages, lack of adjustment for BMI, and other confounders such as smoking, maternal age, and parity.

Kuzmicki et al. discovered that significantly higher plasma resistin between 11 and 13 weeks of pregnancy existed in participants, who later developed GDM as compared to their healthy control counterparts, but this finding is contrary to a report of Megia et al., which indicated that GDM is associated with lower resistin levels [125,126].

Importantly, common and sometimes inevitable phenomena during pregnancy such as increased BMI and substantial weight gain are known to cause changes in the metabolism and gene expression of adipocytes, which results in increased lipolysis and release of pro-inflammatory cytokines that recruit and activate macrophages. Macrophages in turn produce large amounts of proinflammatory mediators, including tumor necrosis factor (TNF)-alpha, interleukin (IL)-1, and resistin, that therefore create a positive feedback loop since an insulin-resistant state will be induced and amplified by these inflammatory mediators. Owing to this fact, the hypothesis of the inflammatory pathway having a noticeable effect on glucose regulation during pregnancy is consistent with the potential role of this same pathway in the development of GDM, since pregnancy is an insulin-resistant state [119].

Factors such as advanced maternal age, obesity, family history of diabetes mellitus, history of a poor obstetric outcome, as well as abnormal lipid levels, especially triglycerides, are said to contribute to the development of GDM. Thus, the etiology of GDM is very multilayered, and we should not overlook and underestimate any of those factors.

In summary, it is too early to draw solid conclusions due to the heterogeneity between various studies and the numerous contradictory results. There is a need for more well-designed, high-quality studies to clarify the possible implications of differences in resistin levels in GDM patients compared with healthy pregnant women with similar characteristics. Only then will it be possible to gain a better understanding of the role of resistin in the pathophysiology of GDM.

#### 3.4.2. Preeclampsia

Data concerning the changes in serum resistin levels in preeclampsia are both limited and conflicting. Women with preeclampsia very often have to give birth significantly earlier (37.0 (33–41) weeks) than healthy controls (40.0 (38–41) weeks), and fetal birth weights are significantly lower in the preeclamptic group than in the healthy third-trimester individuals [127]. Higher resistin concentrations in women affected by PE have previously been reported by Haugen et al., however, after controlling for insulin resistance (IR), such findings lost their significance, probably due to unchanged resistin placental gene expression. Higher levels of resistin, in that case, should be attributed to altered renal function in preeclamptic patients [128].

Nanda et al. demonstrated that maternal serum resistin concentrations are increased, at 11 to 13 weeks’ gestation, in pregnancies that subsequently developed PE [121]. Seol et al. discovered that there was a marked elevation in serum resistin levels in women with preeclampsia compared to women with normal pregnancies. However, there were no significant differences in placental expression between normal pregnancies and those complicated with preeclampsia [129]. Therefore, according to those results, increased serum resistin levels in women with preeclampsia might not be assumed to be related to placental production. 

In contrast to the findings of Nanda et al. and Seol et al., the circulating resistin levels were found to be lower in preeclamptic women than those noted in healthy pregnancies by Cortelazzi et al. and Chen et al. Smaller size of the placenta and subsequent reduced placental resistin production could possibly be the reason for those findings. Finally, Hendler et al. failed to find a difference in serum resistin concentrations between pregnant women with and without PE [91].

Chen et al. have also reported that resistin was detected in the trophoblast culture supernatant, which confirms the production of resistin by the human placenta. Therefore, it may be hypothesized that the decrease in resistin levels in women with PE is due to the smaller size of the placenta, but we could not exclude the possibility of lower expression of resistin by the placenta in preeclampsia, as well as the PE responsibility for an exaggeration of insulin resistance [114]. 

It is important to note that in the control and PE pregnancies, there were not any significant correlations between serum resistin and maternal weight, maternal BMI, maternal age, or birth weight, which suggests that the maternal serum concentration of resistin is not defined by the metabolic status of the mother [127].

In short, resistin concentrations in PE may be increased, decreased, or not significantly changed in comparison to those in healthy controls. Such significant discrepancies between these studies arise probably from the heterogeneity of studied populations, different definitions of PE, and the complexity of the disease itself. Therefore, the exact role of resistin in PE still remains unclear.

#### 3.4.3. Other Gestational Complications

The relatively high concentrations of resistin in umbilical plasma samples might suggest a potential role of resistin in the control of fetal energy homeostasis, and intrauterine deposition of adipose tissue, as reported by Cortelazzi et al. and Briana et al. [79,130]. As a result, high circulating resistin levels may enhance hepatic gluconeogenesis, and as a consequence, reduce the incidence of neonatal hypoglycemia. Some factors that affect normal childbirth such as inordinately fat or large fetus may be avoided by negative feedback signaling from adipocytes via limiting excessive proliferation and accumulation of fat tissue in the infant. This adaptive response not only decreases the risk of fetal macrosomia but also may possibly ensure the survival of the newborn by providing adequate glucose amount for central nervous system utilization and subsequently, prevent hypoglycemia at birth with all of its negative consequences [79,130].

However, this result has been contradicted by Wang et al., who displayed a decrease in resistin concentrations both in the maternal and umbilical serum as opposed to the control group. Furthermore, umbilical resistin levels, BMI, and neonatal birth weight show a negative and statistically significant correlation between each other. This supports the notion that resistin has a direct effect on regulation of fetal development and may be related to the occurrence of fetal macrosomia [131].

Kim et al. found evidence of resistin having an inhibitory effect on adipose conversion. Accordingly, it is speculated that resistin may be a feedback regulator of adipogenesis and a signal to decrease and restrict adipose tissue formation [132]. Thus, the lower resistin level discovered by Wang et al. may lose the inhibitory effect on adipogenesis and conversely result in excess adipose tissue formation in fetus. One should keep in mind, however, that a decline of resistin may entail an increase in insulin sensitivity, which results in a much greater glucose uptake in muscle and adipose tissue. Hence, the above-mentioned mechanisms are intertwined with one another and may potentially provide a valuable insight into the relationship of low resistin levels with fetal macrosomia [131].

Umbilical cord blood concentrations of resistin might differ between IUGR fetuses and AGA controls. Briana et al. challenged this hypothesis by demonstrating no significant differences in cord blood resistin concentrations between IUGR cases and AGA controls [130]. Briana’s results suggest that resistin may not be directly involved in the regulation of insulin sensitivity and adipogenesis in the perinatal period.

Furthermore, altered regulation of adipocytokines secretion in the IUGR pregnancies and macrosomic fetuses may be predictive of the occurrence of metabolic diseases in adulthood. A deeper understanding of how early programming of adipose tissue influences postnatal metabolic pathways involved in the development of obesity will support the establishment of more effective preventive strategies and therapeutic approaches to slow down the worldwide epidemic of type 2 diabetes and obesity [130].

### 3.5. Irisin

Irisin is a novel secreted myokine, encoded by the fibronectin type III domain-containing protein 5 (FNDC5) precursor gene. Functional studies of irisin demonstrated that its metabolic role was to mediate exercise-related energy expenditure by turning white adipose tissue (WAT) into brown adipose tissue (BAT) in response to activation of the peroxisome proliferator-activated receptor-gamma coactivator-1 α (PGC-1α) [133]. Irisin is cleaved and secreted mostly from skeletal muscle after exercise, but low levels may also be found in the pancreas, liver, and adipose tissue [133,134,135,136]. Irisin is expressed in the female reproductive system, including the ovary, as well as in the placenta and in neonatal cord blood serum [137,138].

In healthy eumenorrheic women, serum irisin levels vary throughout the menstrual cycle, being higher in the luteal phase than in the follicular phase, with an approximately 26% increase of these levels. The rise of irisin in the luteal phase suggests that it might be involved in the ovulation cycle. Furthermore, serum irisin levels were found to rise during normal pregnancy. Additionally, irisin levels are higher in middle and late pregnancy with respect to early pregnancy in healthy women, with an increase in levels of approximately 16% and 21%, respectively [137,139].

#### 3.5.1. Gestational Diabetes Mellitus

Cui et al. presumes that the low irisin levels may contribute to increased serum glucose, decrease in insulin sensitivity, and increasing insulin resistance after discovering significantly lower levels of irisin in the GDM patient group in comparison with the control pregnant group. Interestingly, this fact may also be supported by animal studies that have shown that exogenous administration of irisin fundamentally decreased blood glucose levels at 60, 90, and 120 min, which is possibly due to irisin’s positive effects on insulin sensitivity and improvement of insulin resistance [140]. Erol et al. also have come to this conclusion after detecting significantly lower circulating levels of irisin in the first trimester and the second trimester in the pregnant woman who subsequently developed GDM, which is suggestive of maternal irisin being possible an effective predictor of the GDM development [141].

A cross-sectional study by Kulhan et al. also showed a lower serum concentration of irisin in pregnant women with GDM when compared with that of pregnant women without GDM. However, the authors additionally discovered that the maternal serum level of irisin has a negative correlation with BMI and a positive correlation with insulin resistance, and was negatively correlated with BMI but positively correlated with insulin resistance [142].

The meta-analysis conducted by Cui et al. found that the irisin levels in the postpartum GDM group varied with the time of collection. No significant difference in circulating irisin was found within 24 h after delivery for GDM and control groups. Then, at two weeks and six weeks postpartum, irisin concentration in the GDM group was significantly lower than in the control group. Subsequently, at three months postpartum, there was no significant difference in blood irisin levels between the GDM group and the control group. However, a follow-up study found that the blood irisin levels were significantly higher for the GDM group than that for the control group, with a median postpartum time of 4 years [140]. Park et al. proposed the concept of compensatory hyperirisinemia, and noted that high levels of irisin were associated with increased risk of metabolic syndrome and cardiovascular disease [143].

Additionally, the meta-analysis by Cui et al. investigates the irisin levels not only in the maternal blood but also in the cord blood and breast milk. Unsurprisingly, the breast milk of the GDM patients had lower concentrations of irisin that of the control group, regardless of colostrum or mature milk [140]. Additionally, Fatima et al. speculated that unfavorable effects on health and lipid regulation may stem from continuous feeding of breast milk with fewer irisin [144]. The remarkable connection between low irisin levels in breast milk and infant weight require further investigation.

#### 3.5.2. Preeclampsia

Garcés et al. showed that irisin levels were lower in pregnant women with preeclampsia compared with normal pregnant women from the early stages of pregnancy. The difference was significant in the third trimester, close to 49% in the levels of this adipomyokine noted in healthy individuals. It is possible that this decrease in serum levels of irisin could be explained in part by the reduction in the contribution of placental and other irisin-secretory tissues in the group of preeclamptic patients. Although the results failed to be significant in this study, the low levels during the first and second trimesters might be a valuable predictor of the development of the disease [137]. In another study, Foda et al. reported that serum irisin levels in normal pregnancies, early in labor, were significantly higher than in mild preeclampsia, which is in agreement with the results of Garcés et al. [137,145].

Interestingly the increased endothelial permeability, in preeclampsia, may be associated with decreased endothelial nitric oxide synthase expression and activity in endothelial cells resulting in vasoconstriction [146]. According to Xiang et al., circulating irisin levels were positively associated with endothelium-dependent vasodilatation in newly diagnosed type 2 diabetic patients without clinical angiopathy, which creates a possible correlation between lowered levels of resistin in the first or second trimester and subsequent development of PE [147].

Foda et al. also compared serum irisin levels in patients with mild PE undergoing vaginal delivery and cesarean section. In patients who experienced vaginal delivery, irisin levels were significantly higher both early in labor and after fetal delivery in comparison to those who underwent cesarean section [145]. Vaginal delivery, like muscular exercise, is associated with a massive release of irisin during the labor from the placenta, or from the uterine and pelvic muscles. These findings are comparable to the previous reports done by Lee et al. and Kurdiova et al. that mention a three-fold increase in serum irisin levels measured immediately following one hour of moderate exercise [135,148]. It could be concluded that labor is a strong stimulus to the release of irisin into the fetal circulation.

In the absence of more evidence, it is only possible to speculate on the mechanism of decreased maternal irisin levels in preeclamptic pregnancies. As to whether alterations in the expression and secretion of irisin-secretory tissues that are compromised in preeclamptic women remains to be elucidated.

#### 3.5.3. Other Gestational Complications

Çaǧlar et al. compared irisin levels in maternal and umbilical cord blood in normal pregnancies and those complicated by FGR. Maternal and umbilical vein irisin levels did not differ significantly between controls and study groups, but umbilical artery irisin levels were significantly lower in the study group. Partly, this finding may be explained by the fact that infants affected by FGR have different body composition with reductions in the levels of body fat and lean mass. One can speculate that it raises the suspicion of low levels of irisin in idiopathic FGR contributing to the pathogenesis of this condition [149]. Furthermore, fetuses with FGR are at higher risk of developing obesity, hypertension, hypercholesterolemia, cardiovascular disorders, glucose intolerance, and type 2 DM at later ages, compared to the general population [150].

Pavlova et al., using a univariate model, discovered a positive association between cord blood irisin concentration and preterm birth occurrence in the studied cohort. A higher irisin level in preterm infants compared with term deliveries was discovered. It could be described by the fact that the onset of labor provides a strong stimulus for the release of irisin into maternal and fetal circulations [145,151], and could increase cord blood irisin level by nearly 40% [152]. In addition, it has been suggested that increased irisin release into cord blood may be caused by temporary uteroplacental ischemia during vaginal delivery, thus leading to fetal stress [145].

Moreover, maternal and neonatal irisin precursor gene FNDC5 polymorphism is associated with preterm birth. Women and neonates bearing the FNDC5 rs726344 GG genotype had a 2.18-fold and 2.24-fold higher chance, for term delivery respectively, compared to both AG and AA genotypes [139].

### 3.6. Omentin

Omentin was initially described in intestinal Paneth cells and has been implicated in the gut defensive mechanisms against pathogenic bacteria [153]. There are two homologs, omentin-1 that is the major circulating form, and omentin-2. This adipokine is preferentially produced and secreted by visceral adipose tissue (VAT) and is predominantly expressed in VAT stromal vascular cells [153]. Yang et al. noted that in vitro experiments revealed that omentin enhances insulin-stimulated glucose uptake in human adipocytes and presents insulin-sensitizing properties [154]. Omentin is also expressed in the heart, lungs, ovary, and placenta. Omentin-1 was shown to be downregulated by insulin and glucose, resulting in decreased levels in overweight women suffering from polycystic ovary syndrome [155]. Moreover, decreased omentin-1 levels were found in patients suffering from obesity and diabetes [156]. Briana et al. also reported that omentin is detectable in the serum of infants and it is mainly derived from fetal and/or maternal tissues. Thus, a positive correlation between maternal and fetal omentin-1 concentrations was found, implying a transplacental transport of this adipocytokine. In addition, the placenta most probably does not contribute to circulating concentrations of omentin-1, as its concentrations do not decline after placental elimination. It was also demonstrated that there are relatively high concentrations of omentin-1 in umbilical serum samples. Therefore, omentin-1 is hypothesized to play a similar role in energy homeostasis [157]. It is worth keeping in mind that glucose acts as a main source of energy during prenatal development and growth. Insulin in turn is well-known for increasing the uptake of circulating glucose by fetal muscle and adipose tissue. Therefore, despite the fact that there is no available information about a potential role of omentin-1 in fetal growth, enhanced growth-promoting effect through its insulin-sensitizing action may be attributed to high levels of omentin-1 in the fetus. 

#### 3.6.1. Gestational Diabetes Mellitus

Overall, a substantial number of studies on the association between concentrations of omentin-1 and DM found contradictory results. For example, in one cohort, similar omentin-1 concentrations between patients suffering from GDM and controls have been observed [158], whereas another cohort showed decreased concentrations in GDM [159].

According to Barker et al. and Franz et al., their prospective case-control studies showed lower omentin-1 levels in obese women, but not in women with GDM [158,160]. Furthermore, they showed a decrease in omentin-1 levels throughout pregnancy, which at first may seem counterintuitive due to the fact that the placenta has been shown to additionally produce omentin-1. Such results may be explained by an increased clearance in the later stage of pregnancy or physiological hemodilution during gestation. However, this needs further evaluation. What is more, De Souza Batista et al. demonstrated that omentin-1 is downregulated by insulin and glucose [161]. It may be assumed that increased insulin resistance during pregnancy could explain decreased omentin-1 levels in the third trimester in both women with GDM and the normoglycemic ones.

Increased risks of obesity, metabolic syndrome, and type 2 diabetes with substantially lower omentin-1 levels and higher serum C-peptide levels in later life have been seen in the offspring of mothers who suffered from GDM [162,163,164]. This could reflect an already altered metabolic state possibly associated with a greater risk of adverse metabolic sequelae in childhood and adult life. Catli et al. supported this statement in their study while demonstrating that omentin-1 is significantly lower in obese children compared to normal-weight ones [165].

Significantly lower omentin-1 concentrations were observed in people with GDM or T2DM than in the controls by Pan et al., whereas no difference was found between the T1DM and controls [166]. Decreased omentin-1 concentrations may be an important indicator of gestational diabetes mellitus and type 2 diabetes mellitus.

The results of the meta-analysis carried out by Sun et al. found that circulating omentin was lower in GDM patients than in controls. This supports the idea of omentin’s chance of being a novel biomarker for the early diagnosis of GDM, which affects many pregnant women [167]. The decreased omentin levels in mothers with GDM, compared with healthy controls, may result from impaired synthesis or release, but the mechanism for this requires further investigation.

#### 3.6.2. Other Gestational Complications

To date, few studies have examined omentin-1 during pregnancy. GDM and omentin levels correlation has been getting the most attention in the last 5 years. Therefore, further investigation is needed to assess the input of omentin into the pathophysiology of other gestational complications.

Šplíchal et al. in their pioneer study concluded that circulating maternal omentin-1 levels were higher in normal spontaneous term births than in preterm births and almost identical in an additional subgroup comparison conducted between preterm births with and without preterm premature rupture of membranes (PPROM) [168]. These results suggest that omentin-1 may play a role in the pathophysiology of preterm birth, but it is not involved in the mechanism of PPROM. Further studies are needed to evaluate the role of omentin-1 in preterm births.

### 3.7. Chemerin

Chemerin is a hormonally active protein that is suspected to be associated with a range of metabolic, inflammatory, and cardiovascular diseases [169,170]. Chemerin is expressed in various human tissues, and also in the placenta. However, its expression is mainly pronounced in the liver and subcutaneous and visceral adipose tissue. [171,172,173]. Its concentration in peripheral blood correlates well with BMI and obesity [174]. Pregnancy is a state of increased chemerin secretion, which rises throughout the gestation [171,175,176]. Nonetheless, its involvement in the regulation of physiological pregnancy is not fully elucidated yet. However, it is thought that chemerin may play a significant role in the pathophysiology of various gestational complications.

#### 3.7.1. Gestational Diabetes Mellitus

Chemerin is known as a novel adipokine thought to have a substantial impact on glucose and lipid metabolism. That effect occurs especially during the pregnancy when the significant increase in chemerin levels is highly pronounced [171,176]. Surprisingly, there was not any correlation between chemerin levels and fasting glucose, C-peptide, and OGTT test results [177,178]. The analyses of relationships between chemerin, fasting insulin, and HOMA-IR (homeostatic model assessment of insulin resistance) values lead to contradictory results. Whereas some authors did not observe any significant correlations between those parameters [177], the others found that chemerin values were positively markedly correlated with HOMA-IR or insulin levels [172,179,180,181]. Chemerin values were also found to be positively correlated with fatty acid-binding protein 4, interleukin-6, and tumor necrosis factor-α [182]. 

**Figure 2:**
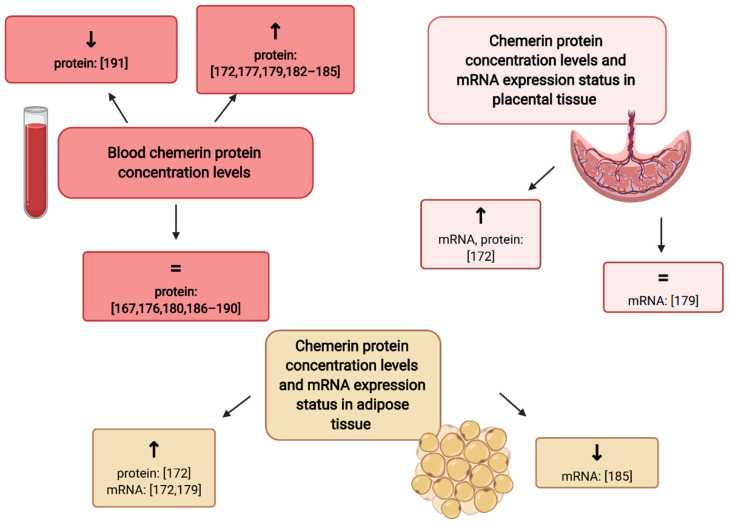
The graphical summary of data concerning chemerin concentration and chemerin mRNA expression levels in GDM patients as compared to control patients. ↑ increase in parameter level as compared to control [172,177,179,182,183,184,185]; ↓ decrease in parameter level as compared to control [191]; =: no significant alterations observed as compared to control [167,176,180,186,187,188,189,190] (Created with BioRender.com).

To analyze its utility in the prediction of GDM, it is crucial to evaluate its blood concentrations in patients with GDM and healthy individuals. Some authors found that chemerin concentrations in peripheral blood are significantly increased throughout the pregnancy in patients with GDM [172,177,179,182,183,184,185]. Whereas some studies reported that chemerin levels are also significantly elevated in the cord blood samples [172,186], the others did not find any differences [187]. Besides the adipose tissue, the expression of chemerin mRNA was also detected in the placental tissue [172,179]. There is a 6- to 24-fold increase in its expression in visceral and subcutaneous adipose tissue, compared with the levels detected in placental tissue samples. It was discovered that the relative chemerin mRNA and protein expression was markedly increased in both placental and adipose tissue samples in diabetic patients [172]. However, Tsiotra et al. did not find any differences in the placental expression of chemerin mRNA. They found significant differences in the expression of chemerin mRNA, in visceral adipose tissue, between the groups of obese-GDM patients and non-obese euglycemic controls. Surprisingly, Li et al. reported that the expression of chemerin mRNA was significantly downregulated in women with GDM, especially in obesity [185]. Other studies did not find any differences in chemerin levels determined in peripheral blood in patients with GDM and healthy controls [176,180,186,187,188,189,190]. Interestingly, Hare et al. noted that the mean third-trimester serum chemerin values, in the healthy control group, were significantly higher than those observed in patients with GDM [191]. Finally, the results of the most recent meta-analysis (20 studies) suggest that there is no difference in circulating chemerin levels in patients with GDM compared with normal pregnancies [167]. The graphical summary of relationships between chemerin concentrations and chemerin mRNA expression according to the diabetic status is presented in Figure 2. A long-term observational study found no differences in chemerin levels 3-years postpartum in women with GDM and controls [192].

Nonetheless, it is noteworthy that first and second trimester multiple regression models revealed that chemerin values are positively associated with the elevated risk of GDM, and along with other parameters, chemerin may be treated as an independent risk factor of GDM [177,182,184,193]. The implementation of the results of chemerin levels measurement improves the predictive values of models used in the prediction of GDM [177,182]. Finally, it is unlikely that its concentration measurement could be used as a single early marker in the prediction of GDM. Performing the ROC (receiver-operator curve) analysis, it was estimated that its capacity to predict GDM was poor or moderate, its accuracy (AUC values) ranged from 0.59 to 0.82 [182,188]. Fatima et al.’s estimated value of chemerin AUC (0.97), with the sensitivity of 96% and specificity of 72%, in the diagnosis of GDM, seems bias compared with other studies [184]. 

It has been found that the frequency of the genotypes of chemerin rs4721 gene polymorphism was significantly differentiated among patients with GDM in comparison to healthy controls. Both the dominant and co-dominant version of the rs4721 genotype was associated with the increased incidence of GDM [194]. Moreover, some studies have considered the possible clinical applications of chemerin measurements in the treatment of patients with GDM. It was speculated that adipocytokines concentrations assessment could be used in the prediction of a need of drugs administration, for glycemic control, in the patients who used the nutritional therapy. Nevertheless, there was not any significant connection between poor glycemic control results in those patients and the chemerin levels [195]. 

Chemerin concentrations could also be measured in different body fluids. Okten et al. performed an analysis of adipocytokines concentrations in saliva. Chemerin levels in women with GDM were found to be markedly elevated in comparison to healthy controls [178]. Furthermore, chemerin concentrations in maternal milk were increased compared with values determined in peripheral blood. Mothers with GDM had significantly higher levels in their milk compared with controls [196].

In summary, the potential role of chemerin levels assessments, in gestational diabetes mellitus, was mainly restricted to the prediction of GDM development. Even though some studies suggested its possible usefulness in that indication, its predictive value is insufficient to be applied in standard clinical practice.

#### 3.7.2. Preeclampsia

Even though the pathogenesis of PE is still not fully elucidated, the disturbances in chemerin secretion are suspected to be associated with its increased incidence. Xu et al. reported that first trimester chemerin serum values were significantly elevated in patients who developed preeclampsia after 20 weeks of pregnancy. They proposed chemerin as an independent risk factor of preeclampsia, with the estimated sensitivity of 87.8% and specificity of 75.7%, in the prediction of preeclampsia (threshold value > 183.5 ng/mL, estimated AUC—0.85) [197]. Due to that fact, chemerin may be regarded as a potential early marker of preeclampsia. The patients with preeclampsia had also markedly increased second and third trimester chemerin blood levels compared with normotensive individuals [181,198,199,200]. Furthermore, it was noted that the chemerin concentrations correlated with the severity of the pathology. Patients with severe preeclampsia had significantly higher chemerin blood levels in comparison to those with mild preeclampsia or healthy control group [181,197,199]. Both chemerin mRNA and protein expression were substantially increased in placental tissue samples from patients with preeclampsia [200]. The ROC analysis revealed that chemerin levels assessment has high sensitivity—95.5% and specificity—95.7% in the diagnosis of preeclampsia (threshold > 252 ng/mL, AUC—0.98) [181]. The chemerin levels were found to be significantly correlated with maternal blood pressure values, renal function parameters (proteinuria, creatinine), hepatic parameters (ALT, AST), and other adipokines (leptin and adiponectin) [181,198,199,200]. Maternal obesity additionally promotes increased chemerin production in the preeclamptic pregnancies [201]. Furthermore, the neonatal parameters (birth weight and Apgar score) were found to be related to maternal chemerin levels [181]. Long-term observations discovered that chemerin levels remain significantly elevated, for at least 6 months, after the pregnancy in patients diagnosed with preeclampsia [198].

Those results suggest that chemerin may play a role in the development of preeclampsia. Nonetheless, the possible molecular mechanism of its activity is still unknown.

#### 3.7.3. Other Gestational Complications

Chemerin levels are independently associated with fetal growth and neonatal birth weight [184,202]. Furthermore, it was found that chemerin concentrations in both mixed arteriovenous and arterial cord blood samples were markedly elevated in LGA newborns in comparison to those with normal birth weight [202,203]. In contrast, Van Poppel et al. did not observe any correlation between birth weight and chemerin concentrations in neither amniotic fluid nor cord blood [186]. Interestingly, the analysis of chemerin concentrations in discordant twins revealed that neonates with SGA (small for gestational age) had significantly lower chemerin cord levels than their siblings [202]. The follow-up study performed on the group of SGA newborns demonstrated that newborns with slow catch-up growth had significantly higher chemerin levels, measured at 3 months of age, compared with those with normal catch-up growth [204].

To analyze the regulatory activity of chemerin in the maintenance of early pregnancy, Yang et al. assessed the expression of chemerin and its receptors in the decidua tissues from women with spontaneous and planned selective abortions. They discovered the significantly increased chemerin expression and downregulated expression of its receptors (chemokine-like receptor-1 (CMKLR1)) in patients with spontaneous abortions [205]. The intrauterine injection of chemerin receptors’ antagonist resulted in a higher rate of embryo resorption in mice and lower ERK1/2 phosphorylation rate in uterine tissue [205]. Interestingly, they also described the association between the pre-pregnancy serum chemerin levels and the risk of spontaneous abortion in women with polycystic ovary syndrome (PCOS). According to their results, diminished chemerin levels, in patients with PCOS, may be treated as an independent risk factor of spontaneous abortion [206].

Finally, it was found that prenatal exposure to tobacco may alter the chemerin gene expression in neonates. Reynolds et al. observed a significant increase in chemerin mRNA expression and the reduction in chemerin DNA methylation in the group of newborns born to smoking mothers [207]. 

### 3.8. Apelin

Apelin is a peptide hormone widely expressed in various human tissues. Apelin and its receptors (APJ) are thought to be involved in the regulation of numerous physiological processes [208], making the Apelin/APJ axis a promising target for implementing novel therapeutic strategies for a range of metabolic and cardiovascular diseases [209,210]. It was reported that the state of physiological pregnancy is associated with a significant reduction in apelin secretion [211]. Both the possible applications of apelin measurements and its potential as a new therapeutic strategy during pregnancy will be considered in this section. 

#### 3.8.1. Gestational Diabetes Mellitus

It has been reported that apelin levels, measured in the peripheral blood samples, were significantly elevated in the group of GDM patients and rise throughout their pregnancy [212,213]. However, some authors did not find any differences in apelin levels measured in the maternal plasma or umbilical cord blood [213,214,215]. The expression of apelin mRNA in subcutaneous adipose tissue, visceral adipose tissue, and placental tissue samples was not altered in women with GDM as compared to healthy individuals [214]. The placental apelin mRNA expression and apelin protein abundance were significantly higher (10-fold to 20-fold) than those determined in adipose tissue [214,216]. Furthermore, its expression markedly correlated with the expression of its receptors [214]. On the contrary, the others noted that apelin levels in the peripheral or cord blood samples were lower in patients with GDM [215,217,218]. Also, the apelin concentrations in human breast milk were significantly lower among diabetic patients [218]. However, the results of a reliable meta-analysis suggest that there are no differences in apelin levels in GDM patients and healthy individuals [167]. The summary of data concerning apelin concentration and apelin mRNA expression levels in GDM patients as compared to control patients is shown in Table 1. The ROC analysis revealed that apelin has a lower negative predictive value as a screening tool to exclude GDM as compare with leptin and adiponectin [217]. Most studies did not find any correlations between apelin and BMI and other metabolic parameters such as fasting glucose, insulin, glycated hemoglobin, HOMA-IR, and lipids [213,214,215,216,217]. However, Guo et al. reported that apelin values, in GDM patients, were negatively correlated with total cholesterol levels [212].

The follow-up observational study found that apelin levels were significantly lower among women who experienced GDM in previous pregnancy in comparison to healthy controls. Interestingly, they noted that apelin levels were inversely associated with fasting glucose, OGTT results, and carotid intima-media thickness (IMT) [219]. IMT is a parameter applied in the prediction of the risk of cardiovascular events [220]. Those outcomes support the hypothesis that the reduction in apelin secretion in women with the history of GDM may play a role in their higher susceptibility to the development of T2D and its cardiovascular consequences [221].

In summary, the results of the studies focused on apelin measurements are inconsistent. Nonetheless, based on the currently available clinical date, it is unlikely that apelin could be applied as a marker of glucose intolerance and insulin resistance. It should be highlighted that the highly pronounced local placental apelin expression illustrates its potential para- and autocrine fetoplacental activity during gestation which requires further investigations.

#### 3.8.2. Preeclampsia

It has been established that serum apelin levels were significantly decreased among patients with preeclampsia [222,223,224,225]. Moreover, alternations in apelin levels were also identified between the groups of patients diagnosed with mild and severe preeclampsia [222,226]. Similar differences were also observed in the samples of umbilical cord blood [226]. The placental expression of apelin and its receptor gene mRNA, as well as their protein products, were decreased in samples of placental tissue in preeclamptic women [227,228]. On the contrary, some authors report significantly higher serum apelin levels among preeclamptic women or no difference in apelin levels in patients with mild and severe preeclampsia [227,229,230]. However, there is a strong negative correlation between blood pressure values and apelin levels [222], and a reverse correlation with BMI [224]. The ROC analyses revealed that apelin has poor reliability as a diagnostic tool in the exclusion of preeclampsia—sensitivity (61–64%) and specificity (61–73%, AUC (0.63–0.67)) [224]. 

The immunostaining of placental tissue samples illustrated that the expression of apelin and its receptors (known as APJ receptors) in term placenta was highly pronounced especially in the cytoplasm of syncytiotrophoblast cells of the chorionic villi, while its presence during the early gestation was mainly detected at the cellular membranes of syncytiotrophoblasts and extravillous trophoblast cells, which may suggest their active involvement in the process of early placentation [227]. It was discovered that apelin present in the chorionic villi exists in various molecular isoforms. However, the pyroglutamate apelin-13 ((Pyr1)-apelin-13) is the most predominant form [228]. The potential molecular mechanism of apelin activity and its contribution to the development of preeclampsia may be elucidated via the effects caused by the stimulation of its cellular receptors. It was proposed that deficiency in the placental elabela (ELA) secretion (a protein, ligand of the APJ) may be liable for the abnormal early placentation and as a consequence could lead to the development of PE in the murine model [231]. It was established that ELA has a capacity to increase the trophoblast invasiveness in vitro [232]. The pathogenesis of preeclampsia is also believed to be connected with the vascular endothelial dysfunction [233]. The study performed on the population of human umbilical vein endothelial cells revealed that the treatment with ELA solutions improved their viability, migration, and tube formation abilities, which may be beneficial for the patients with features of PE. Those effects were said to be invoked by the upregulation of the APJ expression and the activation of PI3K/Akt pathways [234]. Interestingly it was noted that elabela blood levels, mRNA, and protein expressions in placental tissue are also decreased in patients with late-onset PE [235]. In contrast, Para et al. found ELA levels to be significantly increased in women with late-onset PE in comparison to healthy individuals [236]. Nonetheless, it is noteworthy that the other observations performed in humans did not reveal any differences in circulating ELA levels and its placental mRNA expression in preeclamptic pregnant women and healthy individuals [237]. Finally, the potential role of ELA-APJ axis in the pathophysiology of human disease remains unclear.

Based on those promising early findings, Yamaleyeva et al. assessed the efficacy and safety of (Pyr1)-apelin-13 administration in transgenic rats with preeclamptic features. Rats treated with apelin derivatives have significantly lower blood pressure, normalized heart rate variability, and increased ejection fraction. Moreover, apelin administration resulted in decreased proteinuria and improved the renal histopathological findings (lower renal cortical collagen deposition and expression of oxidative stress markers), as compared with the control group without any substantial adverse effects [238]. Furthermore, Wang et al. reported that apelin administration in preeclamptic rats reduced the mean maternal blood pressure and the levels of oxidative stress indicators. Notably, they also observed a significant increase in mean fetal weight, embryo survival rate, and the expression of endothelial nitric oxide synthase [239].

Therefore, those experimental studies made the first step in the development of a novel therapeutic strategy for women with preeclampsia. Nonetheless, the clinical translation of those animal outcomes requires further validations.

#### 3.8.3. Other Gestational Complications

Apelin levels measured in neonates were positively correlated with their birth weight, HOMA-IR, and fasting insulin levels [240]. Furthermore, LGA neonates had markedly increased circulating apelin levels. Interestingly, LGA newborns of diabetic mothers have significantly elevated apelin concentrations compared with LGA infants of mothers with normal glucose tolerance [240]. However, there was not any significant correlation between apelin concentrations in maternal circulation and neonatal birth weight [215,216,241], whereas the others found an inverse correlation between those parameters [213]. Vaughan et al. noted that obese mothers delivered newborns with significantly increased birth weight in comparison to those with normal BMI. However, they did not find any relationship between maternal obesity and apelin levels [216]. Furthermore, it was reported that there was not any association between late-pregnancy maternal apelin levels and the incidence of adverse perinatal outcomes (SGA, FGR, preterm deliveries, and admission to neonatal intensive care units) in either preeclamptic patients or healthy pregnant women [224]. In contrast, Van Mieghem et al. reported that they found significantly lower apelin levels in pregnancies complicated with fetal growth restriction [242]. However, the study groups were relatively small. Finally, there is not enough available data to analyze the potential contribution of differences in apelin levels to the development of the fetal growth abnormalities. 

Ratana et al. reported that they observed significantly lower apelin mRNA and protein expression in fetal membranes in patients after the spontaneous labor in comparison to tissues collected after the cesarean section. Moreover, they found the transfection of apelin siRNA into the primary amnion cells promoted the interleukin (IL)-1β-induced release of pro-inflammatory cytokines (IL-6, IL-8) and pro-labor mediators (prostaglandin E_2_, prostaglandin F_2α_) [243]. Their findings could suggest the possible involvement of downregulation in apelin expression in the induction of the spontaneous rupture of membranes and vaginal delivery. Furthermore, it was discovered that apelin has the capacity to inhibit spontaneous and oxytocin-induced uterine contractility [244]. The authors speculated that generally higher apelin concentrations in obese individuals might be responsible for the reduction of uterine contractility in vivo, which put them at increased risk multiple labor complications [244,245]. It was proposed that prolonged fasting could lead to elevation of circulating apelin levels during gestation [246].

## 4. Future Perspectives

It is unlikely that biomarkers such as leptin, resistin, irisin, apelin, chemerin, and omentin will be implemented as novel, reliable, independent early markers of various gestational pathologies because their independent predictive values are generally insufficient. The majority of pregnancy-related complications, including GDM, pregnancy-induced hypertension, or preeclampsia, always occur in the middle or late pregnancy. Thus, there is a possibility that the analyses of adipokines’ concentrations performed, at the early stage of gestation, together with the assessments of standard maternal risk factors of GDM, PE, etc., could improve the early prediction and prevention of those pregnancy complications. Their combined predictive values should be investigated in further studies. Nonetheless, there is an uncertainty whether the alternations in these serum markers happen before or after the clinical manifestation of those diseases. Importantly, it should be highlighted that such factors as advanced maternal age, obesity, family history of diabetes mellitus, history of a poor obstetric outcome, as well as abnormal lipid levels, especially triglycerides, all together are said to contribute to the development of GDM. Thus, the etiology of GDM and other gestational pathologies is multilayered, and we should not overlook and underestimate any of them.

Whereas the recent human studies focused mainly on the analyses of adipokines’ concentrations in peripheral blood and local expression of their encoded genes in both placental and adipose tissue, the animal studies shed new light on the elucidation of their biological properties analyzing the expression of their receptors and intracellular consequences of its stimulation (for example, the Apelin/APJ axis in relation to PE in mice). Further studies in that matter could establish novel targets for innovative therapeutic strategies in various gestational complications in humans.

## 5. Conclusions

Numerous experimental studies established significant alterations in adipokine secretion patterns throughout the normal and high-risk pregnancies. Nonetheless, most studies have conflicting results potentially due to differences in inclusion criteria (age, BMI, or comorbidity, etc.). The alterations in the secretion patterns of the analyzed adipokines illustrate their potential role in the development of physiological adaptations to pregnancy and delivery. Nevertheless, the underlying mechanisms of adipokine involvement in the pathophysiology of pregnancy-related diseases remain unclear. Maternal obesity often independently affects the expression of multiple adipokines in the adipose and placental tissue. Therefore, the relationships between the analyzed adipocytokines and pregnancy-related complications should be interpreted separately referring to maternal pre-pregnancy BMI and gestational weight gain. Several adipokines were proposed as potential novel early markers of heterogeneous gestational complications. Nonetheless, their prognostic value is mostly insufficient to be implemented into standard clinical practice. Routine assessments of adipokine levels during pregnancy is currently not recommended in the management of both normal and complicated pregnancies. Based on the animal models, the future application of adipokines and their placental receptors as new therapeutic targets for pharmacological interventions seems promising but requires further validations in humans.

## Figures and Tables

**Table 1:** The summary of data concerning apelin concentration and apelin mRNA expression levels in GDM patients as compared to control patients.

Characteristics of the Study Group	Measured Data Type	Parameter Level as Compared to Control	References
Analysis of 20 studies concerning apelin serum concentration levels in 1493 GDM and 1488 control patients	protein concentration	not altered	[167]
Apelin peripheral blood concentration levels in 79 GDM and 80 control patients in the second trimester, as well as in 87 GDM and 88 control patients in the third trimester	protein concentration	increased	[212]
Apelin maternal serum concentration levels in 30 GDM and 30 control patients	protein concentration	increased	[213]
Apelin cord blood concentration levels in 30 GDM and 30 control patients	protein concentration	not altered	[213]
Apelin plasma concentration levels in 101 GDM and 101 control patients between 24 and 32 weeks of gestation, as well as in 20 GDM and 16 control patients at term	protein concentration	not altered	[214]
Apelin mRNA expression levels in SAT, VAT, and placental tissue in 20 GDM and 16 control patients at term	mRNA expression	not altered	[214]
Apelin maternal blood concentration levels in 24 GDM and 21 control patients	protein concentration	not altered	[215]
Apelin cord blood concentration levels in 24 GDM and 21 control patients	protein concentration	decreased	[215]
Apelin serum concentration levels in 127 GDM and 109 control patients (during pregnancy), as well as 30 GDM and 20 control patients (post-partum)	protein concentration	decreased	[217]
Apelin serum concentration levels in 10 GDM and 10 control patients	protein concentration	decreased	[218]
Apelin breast milk concentration levels in 10 GDM and 10 control patients	protein concentration	decreased	[218]

Abbreviations: GDM—gestational diabetes mellitus; SAT—subcutaneous adipose tissue; VAT—visceral adipose tissue.
